# Awareness and Knowledge of Pneumococcal Vaccination in Cardiology Outpatient Clinics and the Impact of Physicians’ Recommendations on Vaccination Rates

**DOI:** 10.3390/vaccines11040772

**Published:** 2023-03-31

**Authors:** Tuba Ekin, Mehmet Kış, Fatih Güngören, Onur Akhan, Adem Atıcı, Ayşegül Ülgen Kunak, Deniz Mutlu, Fahrettin Katkat, Mevlüt Demir, İbrahim Saraç, Elton Soydan, Dilay Karabulut, Medeni Karaduman, Çağlar Alp, Lütfü Bekar, Ferit Böyük, Mehmet Şahin Adıyaman, Mehmet Kaplan, İsmet Zengin, Serhat Çalışkan, Tarık Kıvrak, Ahmet Öz, Hayati Eren, Murat Bayrak, Umut Karabulut, Selvi Öztaş, Ramazan Düz, Ömer Uluuysal, Ahmet Balun, Gurur Nar Sağır, Hasan Kudat, Hilal Erken Pamukçu, Özge Özcan Abacıoğlu, Ömer Görkem Göldağ, Çağlar Özmen, Şeyda Günay, Mehdi Zoghi, Asım Oktay Ergene

**Affiliations:** 1 Clinic of Cardiology, Sorgun State Hospital, 66700 Yozgat, Turkey; 2 Department of Cardiology, Faculty of Medicine, Dokuz Eylül University, 35340 İzmir, Turkey; 3 Department of Cardiology, Faculty of Medicine, Harran University, 63000 Şanlıurfa, Turkey; 4 Cardiology Department, Bilecik Training and Research Hospital, 11130 Bilecik, Turkey; 5 Cardiology Department, Faculty of Medicine, Istanbul Medeniyet University, 34722 Istanbul, Turkey; 6 Antalya Private Medstar Topçular Hospital Cardiology Clinic, 07200 Antalya, Turkey; 7 Department of Cardiology, Istanbul Medical Faculty, Istanbul University, 34452 Istanbul, Turkey; 8 Cardiology Department, Bagcilar Training and Research Hospital, University of Health Sciences, 34165 Istanbul, Turkey; 9 Department of Cardiology, Faculty of Medicine, Kütahya Health Sciences University, 43270 Kütahya, Turkey; 10 Department of Cardiology, University of Health Sciences, Education and Research Hospital, 25240 Erzurum, Turkey; 11 Department of Cardiology, Faculty of Medicine, Ege University, 35100 İzmir, Turkey; 12 Department of Cardiology, Bakırköy Dr. Sadi Konuk Training and Research Hospital, 34450 Istanbul, Turkey; 13 Cardiyology Department, Van Yüzüncü Yıl Universty, 65080 Van, Turkey; 14 Department of Cardiology, Faculty of Medicine, Kırıkkale University, 71450 Kırıkkale, Turkey; 15 Department of Cardiology, Hitit University Corum Erol Olcok Training and Research Hospital, 19040 Corum, Turkey; 16 Department of Cardiology, Yedikule Chest Disease and Thoracic Surgery Training and Research Hospital, 34020 Istanbul, Turkey; 17 Department of Cardiology, Gazi Yaşargil Training and Research Hospital, 21010 Diyarbakır, Turkey; 18 Department of Cardiology, Gaziantep University School of Medicine, 27310 Gaziantep, Turkey; 19 Department of Cardiology, Bursa City Hospital, 16110 Bursa, Turkey; 20 Department of Cardiology, Bahçelievler State Hospital, 34476 İstanbul, Turkey; 21 Department of Cardiology, Faculty of Medicine, Elazığ Fırat University, 23119 Elazığ, Turkey; 22 Department of Cardiology, İstanbul Training and Research Hospital, University of Health Sciences, 34098 İstanbul, Turkey; 23 Department of Cardiology, Elbistan State Hospital, 46300 Kahramanmaraş, Turkey; 24 Antalya Kepez State Hospital Cardiology Clinic, 07320 Kepez, Turkey; 25 Department of Cardiology, İstanbul Acıbadem International Hospital, 34149 İstanbul, Turkey; 26 Department of Cardiology, Uludağ University, 16059 Bursa, Turkey; 27 Department of Cardiology, Bandırma Onyedi Eylul University, 10200 Balıkesir, Turkey; 28 Department of Cardiology, Dışkapı Yıldırım Beyazıt Training and Research Hospital, 06110 Ankara, Turkey; 29 Department of Cardiology, Adana City Training and Research Hospital, 01120 Adana, Turkey; 30 Department of Cardiology, Training and Research Hospital, 07400 Alanya, Turkey; 31 Department of Cardiology, Faculty of Medicine, Cukurova University, 01330 Adana, Turkey

**Keywords:** awareness of vaccination, pneumococcal vaccine, prevention of cardiovascular diseases

## Abstract

Aim: We aimed to evaluate the awareness of pneumococcal vaccination (PCV13, PPSV23) in general cardiology outpatient clinics and impact of physicians’ recommendations on vaccination rates. Methods: This was a multicenter, observational, prospective cohort study. Patients over the age of 18 from 40 hospitals in different regions of Turkey who applied to the cardiology outpatient clinic between September 2022 and August 2021 participated. The vaccination rates were calculated within three months of follow-up from the admitting of the patient to cardiology clinics. Results: The 403 (18.2%) patients with previous pneumococcal vaccination were excluded from the study. The mean age of study population (n = 1808) was 61.9 ± 12.1 years and 55.4% were male. The 58.7% had coronary artery disease, hypertension (74.1%) was the most common risk factor, and 32.7% of the patients had never been vaccinated although they had information about vaccination before. The main differences between vaccinated and unvaccinated patients were related to education level and ejection fraction. The physicians’ recommendations were positively correlated with vaccination intention and behavior in our participants. Multivariate logistic regression analysis showed a significant correlation between vaccination and female sex [OR = 1.55 (95% CI = 1.25–1.92), *p* < 0.001], higher education level [OR = 1.49 (95% CI = 1.15–1.92), *p* = 0.002] patients’ knowledge [OR = 1.93 (95% CI = 1.56–2.40), *p* < 0.001], and their physician’s recommendation [OR = 5.12 (95% CI = 1.92–13.68), *p* = 0.001]. Conclusion: To increase adult immunization rates, especially among those with or at risk of cardiovascular disease (CVD), it is essential to understand each of these factors. Even if during COVID-19 pandemic, there is an increased awareness about vaccination, the vaccine acceptance level is not enough, still. Further studies and interventions are needed to improve public vaccination rates.

## 1. Introduction

The major causes of death worldwide are cardiovascular illnesses. Management of risk factors and efficient treatment are key to lowering the burden of illness [1]. Acute cardiovascular disorders are believed to be exacerbated by inflammation [2,3]. Acute respiratory infections are recognized to be a trigger of cardiovascular events since infectious illnesses are one of the primary sources of inflammation [4,5]. The World Health Organization (WHO) reported that lower respiratory infections are also among the top causes of death in the world, and patients with chronic cardiovascular diseases (CVD) such as chronic heart failure, coronary or peripheral artery disease, and valvular diseases are at significant risk for community-acquired pneumonia (CAP). The annual incidence of CAP in Europe is reported as 0.5–1.1% [6]. The incidence increases with age. Although there is no surveillance study for *S. pneumoniae* in adults in Turkey, according to the 2004 report, pneumonia is ranked fifteenth with a frequency of 1.15% among the first 20 acute and chronic diseases diagnosed by a physician within the last two months, and lower respiratory tract infections are the fifth most common cause of death. In addition, in terms of disability-adjusted life years, it has been observed that these diseases cause morbidity as well as mortality [7]. It is known that CAP is both more common and more severe in patients with CVD than in those without. In individuals with chronic heart diseases, such as congestive heart failure, CVD, and valvular heart disease, the risk for invasive pneumococcal disease (IPD) has been shown to be 9.9 times higher and the risk for community-acquired pneumonia (CAP) to be 3.3 times higher [8]. Additionally, adults hospitalized for pneumonia or sepsis have an increased risk of cardiovascular disease in the first year and up to 10 years after infection [9,10].

Influenza and pneumococcal infections are significant causes of morbidity and death in high-risk individuals and elderly people. Pneumonia is one of the vaccine-preventable diseases. The vaccine has a cardioprotective effect. The eradication of infections and their consequences as well as the alteration of the immunoinflammatory model of atherosclerosis may all play a role in the mechanism behind the cardioprotective effects of vaccination [11]. Despite several studies demonstrating the effectiveness and affordability of the pneumococcal and influenza vaccinations, high-risk people still have low immunization rates.

All people over 65 years of age should receive the 13-valent Pneumococcal Conjugate Vaccine (PCV13) and the 23-valent Pneumococcal Polysaccharide Vaccine (PPSV23), according to the Centers for Disease Control’s Advisory Committee on Immunization Practices (ACIP), in order to reduce their risk, severity, and complications from pneumococcal disease [12]; nonelderly individuals with intermediate risk of pneumococcal infection should receive vaccination with PPSV23 [13] to which the PCV13 vaccination can be added in high-risk cases [14].

Although the vaccination practice in Turkey is compatible with ACIP [15], in Europe, there is broad consensus that those who are at high risk should be immunized, however the particular advice varies from country to country. Only a few countries, like Sweden and the UK, advise vaccination for everyone over 65 [16].

In adults, influenza virus, human metapneumovirus, rhinovirus, parainfluenza, and coronaviruses (SARS-CoV, MERSCoV, COVID-19) can cause CAP. People over the age of 65 and patients with CVD are at high risk for influenza complications. Therefore, influenza vaccination is recommended for this population. 

In this study, we aimed to investigate:

1. Vaccination coverage rates of the patients admitted to outpatient cardiology clinics;
2. Opinions and attitudes of patients about immunization;
3. Reasons for receipt or intention to have vaccines;
4. Importance of physician recommendation for vaccination;
5. Demographic factors associated with receipt or intention to have a vaccine.

## 2. Materials and Methods

This was a multicenter, observational, prospective cohort study. Patients admitted to the cardiology outpatient clinics who were over 18 years of age and agreed to participate in the study were included. Patients from 40 hospitals in different regions of Turkey participated between September 2020 and August 2021.The participants signed informed consent and completed a self-administered questionnaire in the following sections: basic demographic, clinical characteristics, and knowledge and attitude about vaccination. The patients were informed by the physician about the pneumonia vaccine, and after the information, their medical records were followed up with in terms of whether they were vaccinated or not.

Antihypertensive drugs or high blood pressure on exams (>140/90 mmHg), conducted twice for confirmation, were regarded as indicators of hypertension in patients. With a verified digital sphygmomanometer, blood pressure measures were taken in the office. If a patient was using an antidiabetic medication or had fasting blood sugar levels greater than 126 mg/dL, they were deemed to have diabetes. Hyperlipidemia was diagnosed if the patients were taking lipid-lowering drugs or their lipid levels were high according to the hyperlipidemia guidelines.

History of coronary artery disease, heart failure, dysrhythmia, peripheral arterial disease, valvular heart disease (moderate or severe), cerebrovascular diseases, congenital heart disease, chronic obstructive pulmonary disease, malignancy, solid organ transplantation, and renal diseases were questioned and noted after searching the medical records of study subjects.

### 2.1. Ethical Statement

The study was performed in compliance with the Declaration of Helsinki and the ethics committee of Dokuz Eylul Universty Faculty of Medicine approved the study protocol on 13 July 2020 (Approval Number: 2020/16–18).

### 2.2. Statistical Analysis

Descriptive statistics of the obtained data were calculated as mean, standard deviation, number, and % frequencies. The relationship between categorical characteristics and vaccination status was examined by Pearson chi-square analysis. Independent samples *t*-test was used to compare those who were vaccinated and not vaccinated in terms of age. In addition, the relationship between vaccination status and risk factors was also examined with the multivariate logistic regression model, and adjusted effects were determined. The statistical significance level was *p* < 0.05 and the SPSS (ver. 23) program was used in the calculations.

## 3. Results

**Figure 1:**
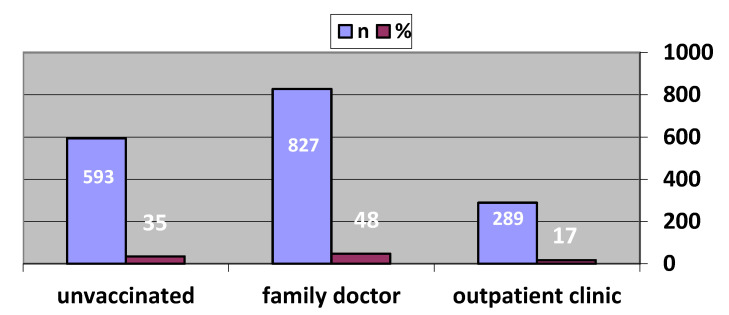
Vaccination preferences of the patients after being informed.

A total of 1808 patients, 44.6% female, with a mean age of 61.9 ± 12.1 years, were included. Only 6.6% of patients were university graduates. The most common coronary risk factor was hypertension (74.1%). The most common CVD was coronary artery disease with a prevalence of 58.7%. (Table 1). Most of the patients (67.2%) reported that they did not know about the pneumonia vaccine. The sources of information on vaccines were mainly TV, internet, and social media. These were followed by medical doctors with a rate of 7.6%. While the majority of the patients (45.2%) stated that they did not have enough information about the pneumonia vaccine, the rate of those who found the vaccine harmful (3.6%) was less than those who found it useful (17.5%). Most of the patients (63.3%) thought that the pneumonia and influenza vaccines were the same vaccine (Table 2). After being informed about the vaccine, during the follow-up, the information on whether 76 of the 1808 patients included in the study had been vaccinated could not be accessed via medical records. Of all patients, 23 patients died due to any cause during follow-up. Vaccination statuses of 1709 patients were determined in the follow-up. The values indicated in bold in Table 3 are made for statistically significant comparisons. Moreover, 1122 patients (66%) preferred to be vaccinated. While 827 patients preferred to be vaccinated at family physicians’ offices, 289 patients preferred vaccination clinics (Figure 1). It was found that when education levels rose, vaccination rates rose as well, but the rate of vaccination among university graduates was not different from those who were illiterate and primary school graduates (Table 3). Among these 1709 patients, 754 patients (44.1%) were over 65 years of age and 484 (64.2%) of these were vaccinated. The majority of patients with any of the diagnoses of CAD, HF, pacemaker/ICD/CRT, CVO preferred to be vaccinated. The comparison of knowledge and attitude about vaccination between vaccinated and unvaccinated patients after being informed about the pneumonia vaccine is summarized in Table 4. The individuals’ intentions to be vaccinated and their conducts were both favorably connected with the doctor’s advice. When the patients were evaluated according to the source from which they obtained information about the vaccine, it was seen that the rate of vaccination was the highest (83.3%) among those who obtained the information from the medical doctor. Multivariate logistic regression analysis showed a significant difference between vaccination and female sex [OR = 1.55 (95% CI = 1.25–1.92), *p* < 0.001], higher education level [OR = 1.49 (95% CI = 1.15–1.92), *p* = 0.002] patients’ knowledge level [OR = 1.93 (95% CI = 1.56–2.40), *p* < 0.001], and their physician’s recommendation [OR = 5.12 (95% CI = 1.92–13.68), *p* = 0.001] (Table 5).

## 4. Discussion

Lower respiratory tract infections cause significant morbidity and mortality in some groups. This effect is more pronounced among adults, particularly those over 65 years of age or those with predisposing health risks. One of the most effective tools that reduces the burden of many infectious diseases is immunization. Despite this fact, adult vaccination rates are frequently far lower than recommended levels, even in industrialized nations [17]. The total vaccination prevalence in the United States was 67 percent among individuals with ASCVD, which is substantially lower than the national objective [18]. In this study we investigated the vaccination rates and the factors affecting vaccine acceptance among the patients who were admitted to outpatient cardiology clinics in Turkey.

Factors affecting vaccine acceptance among patients with CVD were reported as health behavioral factors (preexisting comorbidities, health status, adverse health habits), sociodemographic factors (age, level of education, marital status, level of income, family support), psychological factors (perceived benefit of vaccine, perception about vaccine side effects, knowledge about vaccine), and health access (health insurance, access to vaccine) [19].

### 4.1. Vaccination Coverage by Age Group, Gender, and Education Level

Age plays a significant role in vaccination decisions. Vaccinations are less common among younger persons than among adults over 65. Grandhi et al. showed that persons aged 40 to 64 were more likely to lack immunization than older adults aged 65 or among with ASCVD, with vaccination rates at 54% among adults aged 40 to 64 vs 76% among those aged 65 or older [18] International studies utilizing observational data have demonstrated comparable disparities in influenza vaccination rates according to age, most notably that persons with CVD who were <65 years had lower vaccination rates compared to older groups [20]. In contrast to this, our study results showed that pneumococcal vaccination rates were 66.7 percent for adults aged <65 years compared to 64.2 percent for older people aged >65 years, showing that individuals aged <65 were less likely to be pneumococcal vaccine nonreceivers.

The prevalence of influenza vaccination varies by sex both in the general population and in high-risk populations. According to several researches, women receive fewer vaccinations than men. On the contrary, higher vaccination rates were found in females in some studies [21]. According to our study results, the majority of both males (63%) and females (68.9%) were vaccinated. As a result, the frequency of vaccination of women compared to men was 1.4 times higher (*p* = 0.003).

Although there is a link between maternal education and child vaccination, observational research in Europe found that there is no consistent link between educational attainment and influenza vaccine uptake in those over the age of 14 [22]. Higher educational attainment and vaccination rates were positively correlated in certain nations, such as Austria and Poland, while they were negatively correlated in others, such as Ireland, Italy, and Spain [23]. In our study population, while the pneumoccal vaccination rate was lowest among the uneducated group (57.9%), the highest vaccination rate was seen among high school graduates (75.4%), not among university graduates (62.3%). In our study, the frequency of getting vaccinated was 1.5 times higher in those with primary education than in illiterate subjects (*p* = 0.001). Frequency of vaccination was found to be 2.6 times higher among illiterate people than among those with high education levels (*p* = 0.001). 

### 4.2. Vaccine Perception and Intention to Vaccinate

There is a perception in the general population that the vaccine has no benefit and that there is an increased incidence of vaccine-related adverse events. This affects the vaccine intake negatively. Moreover, one of the main causes of vaccination opposition is vaccine safety concern [21]. In our research group, only 17.5% of the participants thought that the vaccine was beneficial, while 3.6% of the participants thought that the vaccine was harmful.

Adults with CVD who also had a concurrent medical need for the influenza vaccination were more likely to receive it [24]. In addition, many at-risk persons expressed desire to receive vaccinations as compared to those who were not in danger when unvaccinated individuals were asked about their intention to be vaccinated [25]. A review from France revealed that 10% of all people had a risk factor for the pneumococcal illness [26], and according to a French High Council for Public Health research, just 20% of at-risk persons get immunized [27]. In our study results, there was not always a positive relationship between the presence of risk factors and the decision to be vaccinated (Table 3).

One of the most powerful inducers of vaccination uptake was direct advice and instruction from medical personnel [28]. Similarly, our study results showed that 83.3% of those informed by a doctor were vaccinated and the most effective source of information was medical doctors. Frequency of vaccination was found to be two times higher in those who learned about the pneumonia vaccine from a physician compared to those who did not know about it (*p* = 0.010) (Table 5). On the other hand, the frequency of vaccination was found to be 0.2 times lower in those who learned from ‘’other’’ sources (*p* = 0.001). At this point, being informed by physicians had a stronger effect. When asked about opinions about the pneumonia vaccine, the frequency of vaccination was found to be significantly higher than in those who thought it might be harmful in all other answers (Table 5). Knowing that flu and pneumonia vaccines are different did not have a significant effect on the frequency of vaccination.

According to one research, just 8% of adults who had recently been diagnosed with a chronic disease for which pneumococcal immunization was advised received the vaccine in the first year of follow-up, and only 20% did so after five years [25]. Only 7–13% of patients in a German study of individuals with “high-risk” illnesses received a pneumococcal vaccine within three years after diagnosis [29]. These results imply that briefing by a medical professional may be a successful strategy for promoting pneumococcal vaccination, especially among at-risk people [30,31,32]. A self-administered, anonymous survey of doctors showed that 25% of subspecialists and 14% of generalists failed to strongly suggest influenza vaccination [33].

The adoption of the influenza vaccination is significantly hampered by a lack of health insurance [18]. If the vaccine is free to access, people are more likely to think about being vaccinated [34]. Work should be done in this regard. Pneumococcal vaccination of elderly and at-risk persons is cost-saving in Turkey [35]. However, in Turkey, vaccination rates are still low, although patients pay no money to be vaccinated.

Although the vaccination recommendation for patients under 65 years of age with comorbidities and for individuals over 65 years of age is very strong, unfortunately our study results showed that the vaccination rate of those with a comorbidity is lower than those without a comorbidity. The vaccination rates for individuals aged under and over 65 are similar. On the other hand, if the source of information about vaccination is a physician, we see that vaccination rates are higher than other sources of information. Our conclusion here is that there is a need for platforms where physicians will provide information to high-risk groups in terms of vaccination in our country. Arrangements are needed to increase the time to be devoted to patient information during clinical visits. Thus, we believe that the tendency to be vaccinated with the recommendation of physicians will increase. 

Similarly, our research results show that as the literacy level rises, the rate of vaccination also increases. For this reason, efforts should be made to increase the education level of the society, along with lessons that will emphasize the importance of vaccination in basic education. In this study, the vaccination rate of university graduates was not as high as we expected. Although this group may have better access to information, it is thought that they are more open to disinformation.

We believe our study showed a homogeneous distribution because it was multicenter and there was participation in it from all of Turkey. The strength of the study increases due to the number of patients included in the study.

Study Limitations: This study’s use of a cross-sectional methodology, which only permits correlations to be identified rather than causal effects, is a significant disadvantage. A national vaccination record would be perfect for quick and precise vaccine uptake evaluation.

In conclusion, to increase adult immunization rates, especially among those with or at risk of cardiovascular disease (CVD), it is essential to understand each factor. Even if during the COVID-19 pandemic, there is an increased awareness about vaccination, the vaccine acceptance level is still not enough. Futher studies and interventions are needed to improve public vaccination rates.

## Figures and Tables

**Table 1:** Basic demographic and clinical characteristics of the patients.

		Study Population (n = 1808)
Age (years), mean ± SD		61.9 ± 12.1
Age ≥ 65 years, n (%)		678 (37.5%)
Sex, n (%) Male Female		1002 (55.4%) 806 (44.6%)
Educational status, n (%)	None	481 (26.6%)
	Primary school	895 (49.5%)
	High school	312 (17.3%)
	University	120 (6.6%)
Diabetes mellitus, n (%)		630 (34.8%)
Hypertension, n (%)		1339 (74.1%)
Hyperlipidemia, n (%)		854 (47.2%)
Coronary artery disease, n (%)		1062 (58.7%)
Heart failure (LVEF < 40%), n (%)		420 (23.2%)
Pacemaker/ICD/CRT implantation, n (%)		120 (6.6%)
Valvular heart disease, n (%)		459 (25.4%)
Peripheral arterial disease, n (%)		106 (5.9%)
Cerebrovascular disease, n (%)		127 (7%)
Congenital heart disease, n (%)		45 (2.5%)
Chronic obstructive pulmonary disease, n (%)		309 (17.1%)
Malignancy, n (%)		85 (4.7%)
Solid organ transplantation, n (%)		3 (0.16%)
Renal failure GFR < 60 mL/mn, n (%)		196 (10.8%)

**Table 2:** Knowledge and attitudes about vaccination.

Questions			n (%)
Do you know about the pneumonia vaccine?		Yes	593 (32.7%)
		No	1215 (67.2%)
Source of information on vaccines	Medical doctor		137 (7.6%)
	Nurse and another medical staff		83 (4.6%)
	Television, internet, social media		211 (11.7%)
	Friends		128 (7.1%)
	Other		34 (1.9%)
Opinions about the pneumonia vaccine	Pneumonia vaccine is very useful		317 (17.5%)
	Pneumonia vaccine is partially useful		441 (24.4%)
	I don’t know enough		817 (45.2%)
	I don’t think the pneumonia vaccine is effective		168 (9.3%)
	I think vaccines can be harmful		65 (3.6%)
Are the pneumonia and flu vaccine the same?		Yes	1144 (63.3%)
		No	664 (36.7%)

**Table 3:** Comparison of demographic and clinical characteristics between vaccinated and unvaccinated patients after being informed about the pneumonia vaccine.

		Vaccinated (n = 1116)	Not-Vaccinated (n = 593)	All n = 1709	*p*-Value
Age (years), mean ± SD		61.6 ± 11.8	62.3 ± 11.9		0.234
Age, n (%) ≥65 years <64 years		484 (64.2%) 628 (66.7%)	270 (35.8%) 317 (33.3%)	754 942	0.286
Sex, n (%) Male Female		**594 (63%)** **528 (68.9%)**	349 (37%) 238 (31.1%)	943 766	**0.010**
Educational status, n (%)	None	253 (57.9%)	184 (42.1%)	437	**<0.001**
	Primary school	568 (66.7%)	284 (33.3%)	852	
	High school	230 (75.4%)	75 (24.6%)	305	
	University	71 (61.7%)	44 (38.3%)	115	
Diabetes mellitus, n (%) None		380 (63.9%) 713 (66.6%)	215 (36.1%) 357 (33.4%)	595 1070	0.254
Hypertension, n (%) None		824 (65.1%) 274 (66.2%)	441 (34.9%) 140 (33.8%)	1265 414	0.652
Hyperlipidemia, n (%) None		513 (63.8%) 578 (67.1%)	291 (36.2%) 283 (32.9%)	804 861	0.154
Coronary artery disease, n (%) None		**626 (62.2%)** **496 (70.7%)**	381 (37.8%) 206 (29.3%)	1007 702	**<0.001**
Heart failure, n (%) LVEF < 40% LVEF 40%–50% LVEF >50%		258 (65.8%) **211 (53.8%)** **639 (70.7%)**	134 (34.2%) 181 (46.2%) 265 (29.3%)	392 392 904	**<0.001**
Pacemaker/ICD/CRT implantation, n (%) None		**55 (53.9%)** **1041 (66.4%)**	47 (46.1%) 527 (33.6%)	102 1568	**0.010**
Valvular heart disease, n (%) None		260 (63.3%) 836 (66.2%)	151 (36.7%) 427 (33.8%)	411 1263	0.278
Peripheral arterial disease, n (%) None		66 (69.5%) 1027 (65.5%)	29 (30.5%) 540 (34.5%)	95 1567	0.433
Cerebrovascular disease, n (%) None		**63 (54.3%)** **1031 (66.2%)**	53 (45.7%) 526 (33.8%)	116 1557	**0.009**
Congenital heart disease, n (%) None		**18 (50%)** **1080 (66.1%)**	18 (50%) 553 (33.9%)	36 1633	**0.044**
Chronic obstructive pulmonary disease, n (%) None		174 (62.6%) 938 (66.1%)	104 (37.4%) 480 (33.9%)	278 1418	0.253
Malignancy, n (%) None		46 (56.8%) 1057 (66.2%)	35 (43.2%) 539 (33.8%)	81 1596	0.081
Solid organ transplantation, n (%) None		1 (50%) 1064 (65.4%)	1 (50%) 563 (34.6%)	2 1627	0.309
Renal failure, n (%) GFR < 60 mL/mn GFR ≥ 60 mL/mn		**110 (59.1%)** **993 (66.7%)**	76 (40.9%) 495 (33.3%)	186 1488	**0.039**

Values in bold are made for statistically significant comparisons.

**Table 4:** Comparison of knowledge and attitudes about vaccination between vaccinated and unvaccinated patients after being informed about the pneumonia vaccine.

Questions		Vaccinated (n = 1116)	Not-Vaccinated (n = 593)	*p*-Value
Do you know about the pneumonia vaccine?	No	723 (64%)	407 (36%)	<0.001
Source of information on vaccines	Medical doctor	110 (83.3%)	22 (16.7%)	
	Nurse and another medical staff	60 (73.2%)	22 (26.8%)	
	Television, internet, social media	126 (62.4%)	76 (37.6%)	
	Friends	91 (72.8%)	34 (27.2%)	
	Other	10 (31.3%)	22 (68.8%)	
Opinions about the pneumonia vaccine	Pneumonia vaccine is very useful	225 (71.2%)	66 (20.9%)	<0.001
	Pneumonia vaccine is partially useful	301 (68.4%)	117 (26.6%)	
	I don’t know enough	465 (57%)	301 (36.9%)	
	I don’t think pneumonia vaccine is effective	103 (61.3%)	59 (35.1%)	
	I think vaccines can be harmful	25 (38.5%)	38 (58.5%)	
Are the pneumonia and flu vaccine the same?	Yes	679 (63%)	398 (37%)	0.002
	No	441 (70.4%)	185 (29.6%)	

**Table 5:** Adjusted effects of risk factors found to be significant by univariate analysis on vaccination status.

		Adjusted OR	95% C.I. for Adj. OR		*p*
			Lower	Upper	
Sex (RC: male)		1.454	1.132	1.868	**0.003**
Education Level (RC: none)	Primary school	1.574	1.198	2.070	**0.001**
	High school	2.620	1.775	3.867	**0.001**
	University	1.111	0.666	1.854	0.687
Coronary artery disease (RC: none)		0.698	0.551	0.885	**0.003**
Ejection fraction (RC ≥ 40–50)		0.602	0.450	0.805	**0.001**
Cerebrovascular disease (RC: none)		0.660	0.438	0.994	**0.047**
Congenital heaart disease (RC: none)		0.389	0.187	0.810	**0.012**
Source of information on vaccines (RC: none)	Medical doctor	2.037	1.189	3.489	**0.010**
	Nurse and another medical staff	1.091	0.626	1.903	0.759
	Friends	1.365	0.864	2.154	0.182
Opinions about the pneumonia vaccine (RC: harmful)	No idea	3.490	1.984	6.141	**0.001**
	Not effective	2.901	1.559	5.398	**0.001**
	Partially useful	4.880	2.744	8.681	**0.001**
	Very useful	6.425	3.495	11.811	**0.001**

RC: reference category, OR: odds ratio, CI: confidence interval. Statistically significant values are in bold.
